# Interventions for Tobacco Prevention and Control in Humanitarian Settings: A Scoping Review

**DOI:** 10.1093/ntr/ntae135

**Published:** 2024-05-31

**Authors:** Nachiket Gudi, Edlin Glane Mathias, Ansuman Swain, Vanshika Gupta, Elstin Anbu Raj, Sanjay Pattanshetty, Sanjay Zodpey, Helmut Brand

**Affiliations:** Department of Health Information, Prasanna School of Public Health, Manipal Academy of Higher Education, Manipal, India; Department of Health Information, Prasanna School of Public Health, Manipal Academy of Higher Education, Manipal, India; School of Sport Exercise and Health Sciences, Loughborough University, Loughborough, UK; Independent Researcher, Delhi, India; Department of Health Information, Prasanna School of Public Health, Manipal Academy of Higher Education, Manipal, India; Department of Pharmacy Practice, Manipal College of Pharmaceutical Sciences, Mahe, India; Department of Global Health Governance, Prasanna School of Public Health, Manipal Academy of Higher Education, Manipal, India; Department of International Health, Care and Public Health Research Institute – CAPHRI, Faculty of Health Medicine and Life Sciences, Maastricht University, Maastricht, The Netherlands; Public Health Foundation of India, New Delhi, India; Prasanna School of Public Health, Manipal Academy of Higher Education, Manipal, India; Department of International Health, Care and Public Health Research Institute – CAPHRI, Faculty of Health Medicine and Life Sciences, Maastricht University, Maastricht, The Netherlands

## Abstract

**Introduction:**

Tobacco usage is an epidemic as statistics point towards smoking as the second leading cause of death. Populations experiencing humanitarian emergencies may experience a higher propensity for tobacco, alcohol, and other substance abuse disorders. This review aimed to map tobacco prevention and control interventions in humanitarian settings.

**Aims and Methods:**

The search for this scoping review was conducted in six databases and supplemented with a gray literature search. Articles were screened at title-abstract and full-text by two pairs of authors, and data was abstracted by three individuals independently. An adapted diffusion of governance framework is used to discuss the findings.

**Results:**

A total of 26 articles were included from the searches conducted in the databases and gray literature. The interventions targeted all age groups. The documents retrieved from the gray literature search were classified as population-based interventions, as they were not restricted to a particular group of individuals. Interventions were delivered at various locations, using different methods and engaging multiple stakeholders. Interventions assessed were grouped into packaging, labeling, and other policy interventions (pricing and taxes).

**Conclusions:**

There are few tobacco prevention and control interventions in the humanitarian context. The diffusion of governance perspective in implementing these interventions in humanitarian settings provides a cue for inter-sectoral cooperation among different stakeholders and disciplines beyond the health sector. Our review recommends exploring complementarity between the demand and supply-side interventions for tobacco control.

**Implications:**

The scoping review has highlighted various tobacco prevention and control efforts in humanitarian settings. The interventions were delivered using various modes, and yet the burden of smoking is higher among the humanitarian population. Further research may use impact evaluation techniques to assess the impact of these interventions to facilitate the re-design of the implementation approach and policy priorities.

## Introduction

Tobacco is an epidemic, as statistics point to smoking as the second leading cause of death.^1^ According to the World Health Organization (WHO), the tobacco epidemic has resulted in the deaths of more than 8 million annually, with 1.2 million deaths resulting from secondhand exposure to tobacco.^2^ Of the estimated 1 billion smokers globally, 80% of them live in the LMICs. Deaths from tobacco are not immediate and lead to other complications, thus making it a major public health concern.

The scenario further worsens in humanitarian settings owing to their poor governance structures, underdeveloped health systems, and weak regulatory mechanisms, thus making it challenging to implement tobacco prevention and control measures. Humanitarian settings are “a range of situations including natural disasters, conflict, slow-onset and rapid-onset events, rural and urban environments, and complex political emergencies in all countries.”^3^ This review is informed by the United Nations Office for the Coordination of Humanitarian Affairs (OCHA) list of humanitarian settings.^4^ Populations dwelling in these humanitarian emergencies may experience a higher propensity for tobacco, alcohol, and other substance abuse disorders. Since most humanitarian settings are low- and middle-income countries (LMICs), global trends show an increased cigarette consumption.^2^ They are subjected to considerable health and economic complications due to contextual factors prevailing in humanitarian settings. The residents are exposed to high social, physical, and mental stress, leading to the loss of life burden of mental trauma in such war-torn landscapes. Reiss et al.^5^ highlighted the increase in tobacco usage among immigrants to the natives.^5^ Evidence suggests that older adults in the Rohingya Refugee Camps perceived a rise in tobacco use during the COVID-19 pandemic.^6^ Such regions have inadequate resources and knowledge to contain the raging tobacco epidemic.^7^ This facilitates the multinational tobacco companies to potentially impact policies, destabilizing attempts to regulate tobacco and expanding the promotion and accessibility.^8^

Tobacco is wrongly viewed as a coping agent for excess stress and frustration in humanitarian settings. The severity of the health impact it has on primary and secondhand smokers is often not understood by those in distress.^9^ Lack of dedicated financial mechanisms to support human resources, political will, limited external support through aid and workforce, and weak law enforcement activities contribute to poor tobacco prevention and control efforts in these settings.^10^ According to the WHO, “Health in all policies is an approach to public policies across sectors that systematically considers the health and health systems implications of decisions, seeks synergies, and avoids harmful health impacts, to improve population health and health equity.”^11^ This highlights the need for designing holistic policies and stronger efforts that would change the conducive environment for tobacco usage in conflict-affected regions.

Various global, regional, national, and subnational organizations are working towards eliminating the tobacco menace. The prevention interventions are constructed around the “Framework Convention for Tobacco Control” (FCTC). In addition, there are noteworthy efforts in diverse settings for tobacco prevention and control, but the synthesis of these efforts in humanitarian settings is lacking.^9,12,13^ Against this background, we conducted a scoping review to map the tobacco prevention and control interventions in humanitarian settings.

## Materials and Methods

A Scoping Review (ScR) approach was used, and the review is reported according to the Preferred Reporting Items for Systematic Reviews and Meta-Analyses extension for scoping reviews (PRISMA- ScR).^14^ A protocol was established a priori, and a detailed methods section can be accessed elsewhere.^15^

1. Identifying the research question: The literature around humanitarian settings has been emerging. There are studies estimating the burden of tobacco usage among these populations.^5,16^ However, there is a lack of literature on tobacco control policies and prevention interventions in these settings. We use a scoping review approach to understand tobacco prevention and control interventions in humanitarian settings.

**Research Question:** “What are the tobacco prevention and control interventions in humanitarian settings?”

### Identifying the Relevant Studies

We identified the literature using the Population, Intervention, Context, Outcome, and Study Design criteria as presented in Table 1.

**Table 1.** Details the Selection Criteria

PICOS	Inclusion criteria	Exclusion criteria
Population	Studies related to all age groups, gender, and ethnicity, including the general population in humanitarian settings. We included studies conducted on different types of tobacco users (current tobacco users), (smoke or smokeless), former tobacco users (smoke or smokeless), never-tobacco users (smoke or smokeless), and ever-tobacco users (smoke or smokeless)	We excluded studies that did not specify the participants’ age groups and tobacco usage status.
Intervention	Studies that contributed to the control and prevention of the use of tobacco products delivered either in person, using technology, or both.	
Context	Studies conducted in the regions classified as humanitarian settings by OCHA.	In the case of multi-country studies, data were extracted for countries listed by the OCHA.
Outcome	*Primary outcome:* Maintenance and cessation of tobacco use, change in the frequency of/ intensity of and quantity of tobacco (smoke and smokeless) consumption. We also extracted data for other outcomes reported in the study.	
Study designs	We included interventional, observational, participatory, and operational and implementation research designs.	We did not include study protocols, conference proceedings, and studies published in languages other than English.

#### Search Strategy and Searches

Since the WHO Framework Convention on Tobacco Control (FCTC) came into force in February 2005, we restricted our searches from March 2005 until May 2022.^17^ We used the following keywords (informed by previous studies)^5,16,18^ to build the search strategy:

“(Smoke*, tobacco*, cigarette*, nicotine, beedi, bidi, papirosi, dip, chew, snuff, snus, e-cigarette, and ENDS) AND (armed conflict, conflict-affected, conflict, war, refugee, internally displaced, forcibly displaced, asylum, and humanitarian) AND (prevention, cessation, quit, and control) AND (list of countries as per OCHA) AND (study designs as per inclusion criteria).” The search was conducted on PubMed (NCBI), Ovid (Medline), Embase (OVID), ProQuest Health and Medical Complete, CINAHL (EBSCHO), and Web of Science (Clarivate). A gray literature search was conducted to complement the bibliometric database search. A detailed list of websites searched is presented in File S1.

### Study Selection

The screening for the title-abstract stage (Ti-Ab) was conducted independently by two pairs of authors (NG, VG, AS, and EGM). Articles at the full-text stage were screened independently and in duplicate by three authors (AS, VG, and EGM). The decision on selecting studies in case of conflicts was resolved through consensus building and arbitrated by NG.

### Data Extraction and Synthesis

Three authors (EGM, VG, and AS) conducted data extraction independently. NG later verified the data extraction sheet for consistency. Data were extracted for title, objective, study design, age of participants, gender, setting (country), type of tobacco usage, status of tobacco usage, name of the intervention, type of intervention, materials used to deliver the intervention, and outcomes of the interventions (as reported by the study) along with challenges faced during the delivery of the intervention.

A quality appraisal of included studies was not performed as this review aimed to map the tobacco prevention and control interventions and record the challenges. We used tables to aid in the presentation of data, and a narrative synthesis was conducted.

## Results

**Figure 1.**
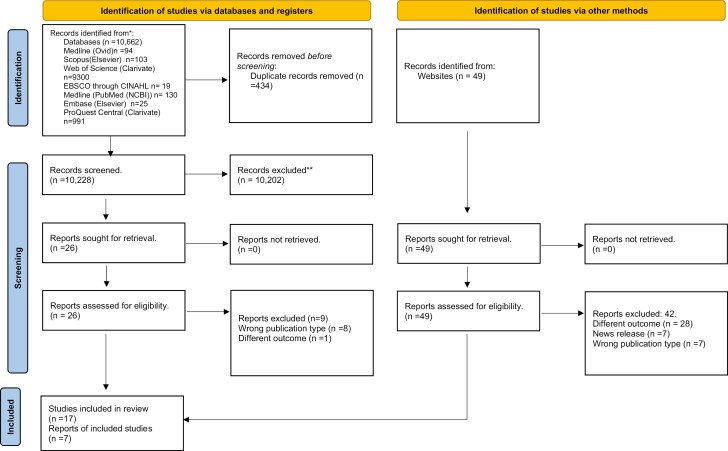
PRISMA flow diagram.

Searches were conducted and updated using Medline (OVID; *n* = 94), Medline (PubMed (NCBI; *n* = 130), Scopus (Elsevier; *n* = 103), Web of Science (Clarivate; *n* = 9300), EBSCO (CINAHL; *n* = 19), EMBASE (Elsevier; *n* = 25), and ProQuest Central (Clarivate; *n* = 991) by NG. We retrieved 10 662 records, removed 434 duplicates using Rayyan.Ai,^19^ and screened 10 228 records at the Title-Abstract (Ti-Ab) stage. We excluded 10 202 records, and 31 articles were deemed eligible for full-text screening. Of these 31 articles, 12 were excluded due to publication type, and two were excluded due to wrong outcomes. We further searched the websites of various organizations for gray literature. We searched 48 web pages and included nine of them for the analysis. We included 17 articles^10,20–35^ from the searches conducted in the databases and nine articles^36–44^ from the gray literature search, including 26 articles for analysis. A PRISMA flow chart represents the number of articles at each stage (Figure 1). Tables S1 and S2 present a list of records excluded during the full-text stage.

The results will be discussed for articles obtained from peer-reviewed literature, followed by gray literature sources (Tables S3, S4, S5, and S6). The results are discussed based on the characteristics of the study (setting, designs, and intervention details), stakeholders engaged, place of intervention delivery, and materials used, and we have grouped the outcomes of these interventions under the following subheadings: (1) Influence of tobacco pack labeling, pictorial warning, and textual warning, (2) Response to policy interventions such as increased pricing and taxes.

### Characteristics of Included Studies

#### Study Settings

One study each was conducted in Syria,^20^ Pakistan,^10^ Cameroon,^32^ India,^34^ Lebanon,^31^ Myanmar,^35^ and Ukraine.^27^ Two studies were conducted in Colombia^22,26^ and Jordan.^21,23^ Three studies^25,30,33^ were conducted in more than one country, and we classified them as “Multiple countries.” Four studies were conducted in Nigeria.^24,28,29^

Seven articles were retrieved through our gray literature search. Four documents^38–41^ covered multiple countries, one^41^ emphasizing the Southeast Asian Region. One document related to Jordan,^37^ the Philippines,^36^ and the Dominican Republic.^44^ Details are provided in Table S3.

#### Study Designs

Studies employed a combination of two or more approaches to counter tobacco issues. Ayub et al. (2015)^21^ used a five-step approach that included forming the “Jordan Tobacco Dependence Treatment Guidelines Group,” conducting a national situation analysis, outlining priorities and content development, national review, and endorsement followed by launch. Ward et al. (2006)^20^ used a combination of household and epidemiological surveys and in-depth ethnographic interviews. Odukoya et al. (2016)^24^ utilized a retrospective record-based approach, but the authors classified it as a descriptive cross-sectional study. Five studies^23,29,31,34,35^ have utilized a cross-sectional survey approach to address their objectives. Uang et al. (2017)^22^ employed key-informant interviews, while Egbe et al. (2018)^28^ used key-informant interviews and social media scanning (newspapers, articles, and written materials). Andreeva et al. (2011)^27^ employed a nationwide omnibus survey. Mapa-Tassou et al. (2018)^32^ employed a qualitative case study design. Girvalaki et al. (2020)^33^ used a quasi-experimental pre-post method. Hussain et al. (2018)^10^ utilized the Randomized Controlled Trial approach. Asare et al. (2019)^25^ and Maldonado et al. (2022)^26^ used secondary data analysis and microsimulation techniques. Perl et al. (2014)^30^ used a combination of rating scales and structured group discussion. Details are provided in Table S3.

#### Place of Intervention Delivery and Materials Used

Interventions were delivered at various locations such as primary care centers,^20^ university campuses,^22^ teaching hospitals,^24^ school classrooms,^10,29^ and cafés.^31^

Various materials have been used to deliver the interventions. Digital projections,^29^ health warnings on packs,^31,34,35^ interactive design applications and distance learning,^20^ signages,^22^ mass-media,^30^ and e-learning curriculum delivered through digital mode.^33^ Among the records retrieved from the gray literature search, nicotine patches,^37^ graphic health warnings,^38,43^ increased schooling,^44^ hiding tobacco products in the stores,^42^ and stronger policy implementation^40^ were observed. Details are provided in Table S4.

#### Intervention Details

Interventions were classified as one-on-one, group, and population-based. Of the 17 included studies, 13 were population-based interventions,^22–27,29–35^ two used a one-on-one approach,^20,28^ and one used a group approach.^10^ Since Ayub et al. (2015)^21^ did not conform to traditional intervention delivery methods, we have designated it “Not classifiable.”

There were various types of interventions, such as the development of guidelines (policy intervention) by Ayub et al. (2015)^21^; the use of pictorial warning labels (PWLs) by Bader et al. (2017)^23^; use of textual and pictorial warnings on tumbac (waterpipe tobacco) boxes by Hallit et al., (2019)^31^; use of health warning labels on Cigarette Packs and tobacco products by Hnin et al., (2020)^35^ and Chopra et al., (2014)^34^; Recall of tobacco pack health warnings by the population in Ukraine^27^; one-one delivery of tobacco cessation interventions in Syria^20^; Tobacco cessation interventions were delivered in tertiary care hospitals^24^; mass-media medium such as radio and television advertisements were used.^30^ Girvalaki et al. (2020) used an innovative e-learning platform.^33^ Behavioral change interventions have been delivered at schools to reduce the habit of smokeless tobacco.^10^ Pricing has been utilized as a policy intervention in Ghana and Nigeria.^25^ A simulation was conducted^26^ to estimate the impact of increasing tobacco taxes. Egbe et al. (2018)^28^ documented the role of various stakeholders in tobacco control. Uang et al. (2017)^22^ focused on monitoring external funding and business support that interfered with adopting and implementing smoke-free policies in Colombia.

Of the 17 included studies, 12 of them targeted their interventions/emphasized their objectives on both males and females.^10,20,23,24,26,27,29–31,33–35^ In contrast, Asare et al. (2019)^25^ included only males in their analysis, and other studies^21,22,28,32^ have used a policy analysis approach and thus have been classified as “Not applicable.” The interventions targeted all age groups, ranging from children to older adults. The documents retrieved from the gray literature search were classified as population-based interventions, as they were not restricted to a particular group of individuals. Details are provided in Tables S4 and S5.

#### Stakeholders Engaged

Various studies engaged multiple stakeholders from Hospitals, Ministries of Health, volunteers from academic institutions and universities; tobacco control organizations and NGOs, staff from local authorities and health staff; Medical anthropologists, psychologists, epidemiologists, behavioral pharmacologists, and physicians and policymakers. Among the individuals involved in delivering the intervention, health worker-led intervention delivery was most prominent at 41% (7/17).^20,22–24,29,31,33^ One record retrieved from the gray literature search highlighted the engagement of policymakers^36^ while we could infer similarly from others. Details are provided in Table S4.

### Outcomes Assessed

#### Influence of Tobacco Pack Labeling, Pictorial Warning, and Textual Warning

The frequency of seeing packet-warming labels (PWLs) and their influence on willingness to quit smoking and the effect of PWLs on quitting smoking were assessed by Bader et al. (2017)^23^ and slightly over one-third of the individuals who reported seeing PWLs (63.1%) expressed that they were influenced to quit smoking. Andreeva et al. (2011)^27^ found that labeling on the front side of the cigarette packets had better recall than the back side by three times (OR 3.37[95% CI: 2.56 to 4.42]). Adebiyi et al. (2016)^29^ found that pictorial warnings invoked fear among adolescents planning to initiate smoking. Images of cancer of the airway and impotence were judged as more effective by those under the age of 15 than by those 15 and over (*p* = .032).

Hnin et al. (2020)^35^ studied health warning labels and found that nearly all the respondents identified both pictorial and text message warnings. Approximately two-thirds of the respondents intended to reduce cigarettes, and almost one-fifth were willing to quit smoking within six months.

Hallit et al. (2019)^31^ established that having many smokers in the workplace was associated with a lower desire to quit (OR = 0.92). They further found that shocking images had a greater effect than textual warnings (ORa = 2.96). Higher intention and motivation (ORa = 2.61) to stop waterpipe smoking were associated with having both pictorial and textual warnings on tumbac packs (ORa = 3.41). Chopra et al. (2014)^34^ found that over 70% of respondents believed in health warnings to raise knowledge about the health risks of tobacco use and to aid in reducing or ceasing tobacco use. Pictorial warnings were found to be better than text warnings. The most effective warning label, the picture of oral cancer (55.6%), followed by a picture of lung disease (32.3%), was effective.

Ethnographic interviews by Ward et al. (2006)^20^ found that smoking waterpipes was viewed as an aesthetic enjoyable experience while smoking cigarettes was viewed as a mundane anxiety-relieving addiction. Odukoya et al. (2016)^24^ concluded that there is poor documentation of tobacco use inquiry in Nigerian hospitals.

#### Response to Policy Interventions Such as Increased Pricing and Taxes

Tobacco control advocates were successful in Nigeria, and this learning could offer some prospects to other developing economies within Africa and other LMICs.^28^ Mapa-Tassou et al. (2018)^32^ found that Cameroon’s tobacco control policy formulation was influenced locally by the social context of NCDs and globally by adopting the WHO FCTC. Various stakeholders, such as the bar owners’ association and hospitality industry, were pivotal in implementing the tobacco control program. Implementing the prevention and control program was easier in big cities and cities with supportive political leadership, whereas it was weak in rural areas.^22^ Asare et al. (2019)^25^ found that higher cigarette prices reduced 30-day cigarette smoking and tobacco use onset significantly in Ghana and Nigeria. Maldonado et al. (2022)^26^ found that tobacco tax hike reduces the number of smokers (from 4.51 to 3.45 MM smokers) and smoking intensity, resulting in a drop in the number of cigarettes smoked in Colombia (from 332.3 to 215.5 MM of 20-stick packs).

Perceived effectiveness, Anti-industry/government support, Commitment to avoiding tobacco (nonsmokers only), and Quitting Preparedness (smokers only) were assessed by Perl et al. (2014).^45^ People dwelling in smaller urban locations and in homes where smoking was permitted gave higher anti-industry ratings (*p* < .05). Younger participants and males gave lower ratings on Commitment to Avoiding Tobacco (all *p* < .05).^45^ Hussain et al. (2018)^10^ concluded that behavioral change interventions enhanced knowledge scores regarding the health hazards of Smokeless Tobacco (SLT) and betel quid. They also observed an increase in in-use perception scores and quit perception scores in the intervention group compared with those in the control group. They further emphasized that these interventions should be tailored and included in school curricula. The ENSP e-Learning platform had a significant influence on intentions to address tobacco use as a priority, documenting tobacco use, offering support, providing brief counseling, giving written material, discussing available medication, prescribing medication, scheduling dedicated appointments to develop a quit plan, and being persistent in addressing tobacco use with the patients (all *p* < .001).^33^ Details are provided in Table S5.

## Discussion

**Figure 2.**
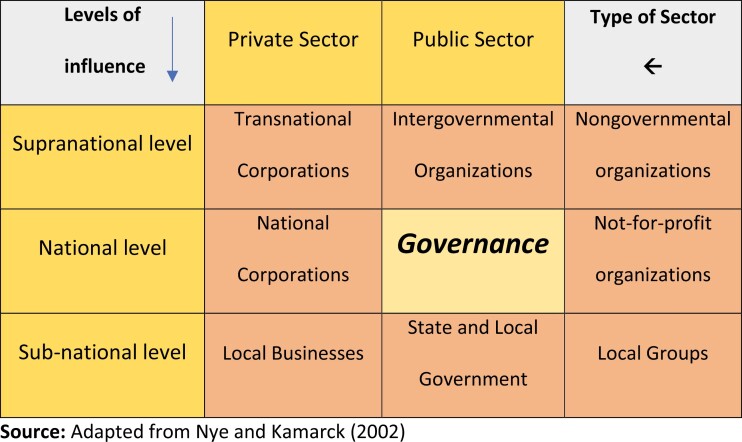
Diffusion of governance framework.

Several barriers exist for the implementation of tobacco control interventions. Areas with humanitarian conflicts lack resources for implementing the entire array of regulatory measures and are known to benefit from regional or subregional initiatives. Inadequate workforce and monetary funding, technical capacity, competing problems, and the lack of political commitment to tobacco control have been quoted across the regions. We have adapted the diffusion of governance framework from Nye and Kamarck (2002)^46^ to discuss the findings considering ongoing efforts (Figure 2).

### Influence of Transnational Corporations and Other Stakeholders

Immense advancement has been made in the struggle against tobacco after adopting the WHO FCTC (WHO FCTC) in 2003. Still, there is a need to expand all government approaches to tobacco control.^38,39^ MPOWER (panel) has been a strategic tool that has helped countries implement guidelines to reduce demand for tobacco as required by the WHO FCTC and to protect their populations from tobacco harm. The WHO FCTC combines measures to reduce demand (price, tax, and non-price measures) for tobacco and the supply of tobacco products, including efforts to prevent interference by commercial and other vested interests of the tobacco industry.^17,47^ Egbe et al. (2018)^28^ found that FCTC ratification has not prevented the tobacco industry from using techniques to stall tobacco control policy in Nigeria.

This study further highlights the role of international funding in assisting tobacco control advocates. A similar observation was noted by Uang et al. (2017).^22^ Another notable example of the tobacco industry overpowering government initiatives was observed in Balanga City, Philippines.^36^

Transnational corporations have often played a pivotal role in preventing or promoting tobacco use.^48^ Our review identified various efforts made by international bodies in humanitarian settings. The Ministry of Health, Jordan, received donations for nicotine replacement therapy from Johnson and Johnson Consumer Health as part of the access initiative for quitting tobacco.^37^ On the contrary, many transnational tobacco companies invest billions and use scarce techniques such as declining employment, harm to government investments, and joining local companies to reap humongous profits.^48^ Some instances further point out that these transnational organizations allot funding to set up smoke-free futures, pump in major investments to alleviate poverty, and coerce governments to amend policies to accommodate the investments. Although transnational corporations invest heavily in creating employment, the quality of life of those involved in preparing tobacco products is often ignored. Evidence suggests that individuals involved in bidi rolling have a high prevalence of respiratory, neurological, and cardiovascular disorders. The evidence further points to perpetual damage, where child workers are prone to low birth weight, stunting, and respiratory and gastrointestinal diseases.^49^

### Importance of Subnational Level Entities

State and local governments are pivotal to implementing national policies, and a similar approach has been observed in Colombia.^26^ Funding from sources beyond health care is crucial to the program. Non-governmental organizations contributed through their technical assistance, and the bar owner’s association provided education campaigns. This highlights the need to engage agencies beyond health in tobacco control efforts, as tobacco is also a trade issue. A similar finding was echoed by Barry et al. (2022),^50^ who highlighted the role of Civil Society Organizations in tackling tobacco industry interference through legal actions in Karnataka, Bihar (India), and Bangladesh. Uang et al. (2017)^22^ observed that the implementation of smoke-free policies was more robust in cities with supportive political leadership than in rural areas and those with limited interests from these groups. This highlights that tobacco prevention and control interventions are multidimensional governance issues rather than unidimensional health issues.

### Engagement of Local Groups

Local groups and community organizations serve as important institutions to implement policies, spread awareness, design interventions, and hand them over to the community once these interventions reach their stage of maturity, as recommended by Laverack et al. (2005).^51,52^ Engaging youth and schoolchildren in tobacco control can pave the way for better tobacco control, as they are highly susceptible.^53^ A study in our review by Jensen and Lleras-Muney in the Dominican Republic found that schooling reduced the likelihood of smoking before 18 years.^44^ Addressing the tobacco smoking issue during the teenage phase and delivering it at schools proved helpful. The Community Intervention Trial for Smoking Cessation (COMMIT) recognized that the active participation of different community organizations was pivotal in changing the tobacco control environment.^54^

### Need for Coordination Among the Intergovernmental Organizations

Globalization, trade, and technological advancement have influenced the promotion of tobacco marketing and consumption worldwide. The global public health community, spearheaded by the WHO and other governmental and non-governmental organizations, has advocated for improved governance to contain tobacco consumption. The landmark treaty, the legally binding instrument, the WHO FCTC (WHO FCTC), paved the pathway for enhanced cooperation among the international community and intergovernmental organizations in tobacco prevention and control.^17^

Even though FCTC advocates for numerous initiatives and innovations in policy regimes to enhance policy coherence among different actors like governmental and non-governmental organizations, significant challenges are posed by broader and uncertain trends in the governance of other sectors like trade, economy, foreign direct investments, and services. These sectors have both direct and indirect impacts on tobacco prevention and control.^55^ For instance, in 2011, the Australian government initiated an essential step by requiring tobacco products to be sold in standard or plain packages to improve tobacco control measures.^56^ However, these measures were challenged before the World Trade Organization because they were inconsistent with the “Agreement on Technical Barriers to Trade” (TBT Agreement) and the “Agreement on Trade-Related Aspects of Intellectual Property Rights (TRIPS Agreement).”^57^

Additionally, the Illegal marketing of tobacco adversely inhibits the countries’ tobacco control efforts. The capability to combat the prohibition of trade differs across countries. The low scores suggest that the LMICs have less potential to tackle the illicit trade. The diminished index score in LMICs was primarily influenced by the lowest scores in tobacco control policies and their practices in trade and customs.^58^ These challenges become daunting in the context of humanitarian situations due to socio-political transition and lack of implementation of tobacco control policies and coherence in inter-sectoral policies. For instance, the Eastern Mediterranean Region has a history of conflict and protracted political and humanitarian crises.^59^ The lack of robust governance structures and implementation policies can be a barrier to executing tobacco control measures and ensuring compliance with FCTC.

The international pledges establish a role for governments and intergovernmental organizations in ensuring human health and well-being by advocating tobacco control. However, the crucial point is that the Tobacco burden is not a single entity or sector/policy issue. Tobacco burden is a multi-sectoral, multi-level, and multi-scalar problem. Due to the nature of the problem, there is a need for inter-sectoral policies that create an ecosystem for collaboration and cooperation among intergovernmental organizations.

### Role of Packaging and Pricing as Policy Tools

The marketing mix of price, place, product, and promotion can be well understood for tobacco, as they are interrelated with the uptake of products. Various regulations have been implemented for tobacco products’ packaging (product and promotion) and marketing (promotion). The studies included in our review further echoed that increasing prices and having warning labels are instrumental in the uptake and utilization of these products. Similar results were found for e-cigarettes in high-income countries.^27,29,31,35^ Evidence from high-income countries suggests that exposure to e-cigarette advertising is positively associated with user status among young people.^60^

The pricing of tobacco-related products needs a regular increase to make it a luxury good, thereby minimizing affordability. Implementing minimum price laws, among others,^61^ could be a favorable approach in a humanitarian setting. Although the tobacco industry has made innovations in its pricing strategies,^62^ it is challenging to sustain them in the long run. High tobacco taxation has raised an additional 12 billion USD in revenue in Afghanistan, which could be allocated to the different budget heads, as banning the industry is often incremental in nature.^63^

### Strengths and Limitations

This review maps the distinct intervention for tobacco prevention and control in humanitarian regions, which is relevant in the current context of achieving SDG 3, holding the commitment of agenda 2030 of “leaving no one behind.”^64^ This research also reflects the need for inclusivity and accountability in addressing the challenges faced in implementation of the tobacco control policies in humanitarian settings. An extensive search was conducted among electronic databases and further supplemented by the gray literature search. Although the gray literature search was comprehensive, we could retrieve only seven articles attributed to limited documentation and presentation of cases in humanitarian settings. We updated the searches once the protocol was published. We revised the data extraction sheet to accommodate comprehensive data extraction, which enhanced the review results; the intervention details were captured in detail and hence, a minor deviation from the protocol is present.

### Directions for Future Research

Future research may focus on the supply-side interventions for tobacco control in humanitarian and other settings, as the scholarly articles are yet to be synthesized. The role of various stakeholders at the local level for tobacco prevention and control using action research approaches may further shed light on the implementation side of these policies. This enables an understanding of social determinants for tobacco control, designing community wide-interventions, and promoting health, thus ensuring sustainability. There are various global and regional policies for tobacco prevention and control. Still, policy to implementation paradigm from the trade lens requires attention as international trade agreements restrict the possibility of establishing new controls.^65^ Understanding these power relations in the humanitarian and LMIC settings offers more profound insights into strengthening prevention and control policies.

## Conclusion

Considering the policy dimensions advocated under FCTC for tobacco prevention and control, this scoping review is valuable to the existing literature. The diffusion of governance perspective in implementing these interventions in humanitarian settings provides a cue for inter-sectoral cooperation among different stakeholders and disciplines beyond the health sector. In addition, this review recommends exploring complementarity between the demand and supply-side interventions for tobacco control.

## Supplementary material

Supplementary material is available at *Nicotine and Tobacco Research* online.

ntae135_suppl_Supplementary_Material

## Data Availability

All the data pertaining to the manuscript is present in the supplementary file.
