# Supplementary material for: Interventions for Tobacco Prevention and Control in Humanitarian Settings: A Scoping Review
Source: Nicotine Tob Res. 2024 May 31;27(1):3–11. doi: 10.1093/ntr/ntae135 (PMC11663804; doi:10.1093/ntr/ntae135)
Supplement: ntae135_suppl_Supplementary_Material [file ntae135_suppl_supplementary_material.docx]

**Supplementary files**

**Supplementary Table 1: List of excluded articles**

| **Sl No** | **Study ID** | **Reference** | **Reason for exclusion** |
| --- | --- | --- | --- |
| 1 | Hoe et al 2021 | Hoe, C., Weiger, C., & Cohen, J. E. (2021). The battle to increase tobacco taxes: Lessons from Philippines and Ukraine. Social Science & Medicine (1982), 279(114001), 114001. doi: 10.1016/j.socscimed.2021.114001 | Case study- different outcome |
| 2 | Nguenha et al 2021 | Nguenha, N., Cunguara, B., Bialous, S., Drope, J., & Lencucha, R. (2021). An overview of the policy and market landscape of tobacco production and control in Mozambique. International Journal of Environmental Research and Public Health, 18(1), 343. doi:10.3390/ijerph18010343 | Publication type |
| 3 | Olowookere et al 2014 | Olowookere, S., Adepoju, E., & Gbolahan, O. (2014). Awareness and attitude to the law banning smoking in public places in Osun State, Nigeria. Tobacco Induced Diseases, 12(1), 6. doi:10.1186/1617-9625-12-6 | Publication type |
| 4 | Erku et al 2019 | Erku, D. A., & Tesfaye, E. T. (2019). Tobacco control and prevention efforts in Ethiopia pre- and post-ratification of WHO FCTC: Current challenges and future directions. Tobacco Induced Diseases, 17(February), 13. doi:10.18332/tid/102286 | Publication type |
| 5 | Krasovsky 2013 | Krasovsky, K. (2013). Sharp changes in tobacco products affordability and the dynamics of smoking prevalence in various social and income groups in Ukraine in 2008–2012. Tobacco Induced Diseases, 11(1), 21. doi:10.1186/1617-9625-11-21 | Publication type |
| 6 | Uang et al 2018 | Uang, R., Crosbie, E., & Glantz, S. A. (2018). Tobacco control law implementation in a middle-income country: Transnational tobacco control network overcoming tobacco industry opposition in Colombia. Global Public Health, 13(8), 1050–1064. doi:10.1080/17441692.2017.1357188 | Publication type |
| 7 | Male et al 2022 | Male, D., Kansabe, S., Lukwata, H., Rubanga, A., Siddiqi, K., Bauld, L., … Dobbie, F. (2022). Smokeless tobacco in Uganda: Perceptions among tobacco control stakeholders. International Journal of Environmental Research and Public Health, 19(6), 3398. doi:10.3390/ijerph19063398 | Publication type |
| 8 | Hoffman et al 2019 | Hoffman, S. J., Poirier, M. J. P., Rogers Van Katwyk, S., Baral, P., & Sritharan, L. (2019). Impact of the WHO Framework Convention on Tobacco Control on global cigarette consumption: quasi-experimental evaluations using interrupted time series analysis and in-sample forecast event modelling. BMJ (Clinical Research Ed.), 365, l2287. doi:10.1136/bmj.l2287 | Publication type |
| 9 | Adugna 2020 | Adugna, A., Azale, T., & Handebo, S. (2020). Seven in every ten khat chewers in Gondar City had an intention to stop khat chewing: cross-sectional study using Transtheoretical Model. BMC Psychiatry, 20(1), 577. doi:10.1186/s12888-020-02984-4 | Publication type |
| 10 | Hoe et al 2021 | Hoe, C., Weiger, C., & Cohen, J. E. (2021). The battle to increase tobacco taxes: Lessons from Philippines and Ukraine. Social Science & Medicine (1982), 279(114001), 114001. doi:10.1016/j.socscimed.2021.114001 | Case study- different outcome |
| 11 | Nguenha et al 2021 | Nguenha, N., Cunguara, B., Bialous, S., Drope, J., & Lencucha, R. (2021). An overview of the policy and market landscape of tobacco production and control in Mozambique. International Journal of Environmental Research and Public Health, 18(1), 343. doi:10.3390/ijerph18010343 | Publication type |
| 12 | Olowookere et al 2014 | Olowookere, S., Adepoju, E., & Gbolahan, O. (2014). Awareness and attitude to the law banning smoking in public places in Osun State, Nigeria. Tobacco Induced Diseases, 12(1), 6. doi:10.1186/1617-9625-12-6 | Publication type |
| 13 | Erku et al 2019 | Erku, D. A., & Tesfaye, E. T. (2019). Tobacco control and prevention efforts in Ethiopia pre- and post-ratification of WHO FCTC: Current challenges and future directions. Tobacco Induced Diseases, 17(February), 13. doi:10.18332/tid/102286 | Publication type |
| 14 | Krasovsky 2013 | Krasovsky, K. (2013). Sharp changes in tobacco products affordability and the dynamics of smoking prevalence in various social and income groups in Ukraine in 2008–2012. Tobacco Induced Diseases, 11(1), 21. doi:10.1186/1617-9625-11-21 | Publication type |

**Supplementary Table 2: List of excluded websites**

| **Sl No** | **Organization** | **Link to paper** | **Reason for exclusion** |
| --- | --- | --- | --- |
| 1 | World Health Organization | <https://www.who.int/news-room/feature-stories/detail/creating-a-smoke-free-city-balanga-city-the-philippines> | Different outcome |
| 2 | World Health Organization | <https://www.who.int/news-room/feature-stories/detail/Johnson-Johnson-donates-nicotine-patches-to-help-thousands-of-jordanians-quit-tobacco-during-COVID-19> | News release |
| 3 | World Health Organization | <https://www.who.int/news/item/09-08-2011-who-urges-more-countries-to-require-large-graphic-health-warnings-on-tobacco-packaging> | News release |
| 4 | World Health Organization | <https://www.who.int/news/item/10-12-2010-world-governments-show-unity-against-tobacco-industry-and-agree-to-new-tobacco-control-measures> | News release |
| 5 | World Health Organization | <https://www.who.int/news/item/19-07-2017-who-report-finds-dramatic-increase-in-life-saving-tobacco-control-policies-in-last-decade> | News release |
| 6 | OCHA | <https://www.thenews.com.pk/print/847811-chest-physicians-demand-increase-in-taxation-on-cigarettes> | Different outcome |
| 7 | UNICEF | <https://www.dawn.com/news/1573905/panah-urges-govt-to-curb-use-of-e-cigarettes> | Different outcome |
| 8 | World Food Program | <https://www.usaid.gov/sites/default/files/documents/1864/210.pdf> | Different outcome |
| 9 | "The United Nations High Commissioner | <https://www.hhs.gov/surgeongeneral/reports-and-publications/tobacco/index.html> | Different outcome |
| 10 | for Refugees" | <https://www.rand.org/news/press/2018/08/06.html> | Different outcome |
| 11 | Médecins Sans Frontières’ (MSF) | <https://www.thenews.com.pk/print/847811-chest-physicians-demand-increase-in-taxation-on-cigarettes> | News release |
| 12 | The Aga Khan Foundation | <https://www.dawn.com/news/1573905/panah-urges-govt-to-curb-use-of-e-cigarettes> | It is a news release |
| 13 | The Aga Khan Foundation | <https://www.usaid.gov/sites/default/files/documents/1864/210.pdf> | It is a news release |
| 14 | United States Agency for International Development | <https://www.hhs.gov/surgeongeneral/reports-and-publications/tobacco/index.html> | Different outcome |
| 15 | United States Agency for International Development | <https://www.rand.org/news/press/2018/08/06.html> | Different outcome |
| 16 | The RAND Corporation | <https://www.rand.org/pubs/external_publications/EP20110070.html> | Different outcome |
| 17 | The RAND Corporation | <https://www.rand.org/pubs/external_publications/EP20090522.html> | Different outcome |
| 18 | The RAND Corporation | <https://www.jahonline.org/article/S1054-139X%2812%2900253-4/fulltext> | Different outcome |
| 19 | The RAND Corporation | <https://www.rand.org/pubs/external_publications/EP66132.html> | Wrong publication type |
| 20 | The RAND Corporation | <https://www.rand.org/pubs/external_publications/EP20100103.html> | Different outcome |
| 21 | The RAND Corporation | <https://www.rand.org/pubs/external_publications/EP67639.html> | Different outcome |
| 22 | The RAND Corporation | <https://www.povertyactionlab.org/evaluation/cares-commitment-savings-smoking-cessation-philippines> | Wrong publication type |
| 23 | The Abdul Latif Jameel Poverty Action Lab (J-PAL) |  | Different outcome |
| 24 | The Gates Foundation | <https://www.fao.org/3/y4997e/y4997e0d.htm> | Different outcome |
| 25 | Food and Agriculture Organization (FAO) | <https://www.fao.org/faolex/results/details/en/c/LEX-FAOC192492/> | Different outcome |
| 26 |  | <https://www.fao.org/3/y4997e/y4997e.pdf> | Wrong publication type |
| 27 | Food and Agriculture Organization (FAO) | <https://www.undp.org/arab-states/publications/jordan-tobacco-control-investment-case> | Different outcome |
| 28 | Food and Agriculture Organization (FAO) | <https://www.undp.org/publications/tobacco-control-governance-sub-saharan-africa> | Wrong publication type |
| 29 | United Nations Development Programme (UNDP) | <https://www.undp.org/asia-pacific/publications/mdg-1-case-study-brief-no-6-tobacco-control> | Wrong outcome |
| 30 | United Nations Development Programme (UNDP) | <https://www.undp.org/publications/policy-brief-benefits-tobacco-control-poor-myanmar> | Different outcome |
| 31 | United Nations Development Programme (UNDP) | <https://www.undp.org/sites/g/files/zskgke326/files/migration/zm/890ad341152566018d2835e19c5ce72ef8e33361c9d1dcab5c7cdb127314cd29.pdf> | Different outcome |
| 32 | United Nations Development Programme (UNDP) | <https://www.undp.org/sites/g/files/zskgke326/files/migration/co/UNDP_Co_PUB_POB_COLOMBIA_FCTC_EN_Jan27_2020.pdf> | Different outcome |
| 33 | United Nations Development Programme (UNDP) | <https://www.undp.org/sites/g/files/zskgke326/files/migration/mm/Myanmar-Tobacco-Control-Investment-Case.pdf> | Wrong publication type |
| 34 | United Nations Development Programme (UNDP) | <https://www.cgdev.org/blog/measuring-effectiveness-health-taxes-cigarette-tax-scorecard> | Different outcome |
| 35 | United Nations Development Programme (UNDP) | <https://www.cgdev.org/blog/health-taxes-save-lives-colombia-version> | Wrong publication type |
| 36 | The centre for Global Development" | <https://www.cgdev.org/blog/tobacco-taxes-win-win-asia-pacific-region> | Different outcome |
| 37 | The centre for Global Development" | <https://www.cgdev.org/publication/meeting-health-challenges-developing-asia-corrective-taxes-alcohol-tobacco-and> | Wrong publication type |
| 38 | The centre for Global Development" | <https://www.cgdev.org/publication/taxing-bads-overview-research-initiatives> | Different outcome |
| 39 | The centre for Global Development" | <https://www.cgdev.org/blog/eliminating-duty-free-tobacco-what-went-wrong> | Wrong outcome |

**Supplementary table 3: Study characteristics**

| **Study ID** | **Title** | **Objective** | **Study design** | **Age** | **Gender** | **Setting** | **Type of tobacco usage (Smoke/smokeless)** | **Status of tobacco usage of the target population (ever users/never users/routine users/former** |
| --- | --- | --- | --- | --- | --- | --- | --- | --- |
| Ward et al 2006 | The tobacco epidemic in Syria | To (1) study tobacco uses and local smoking practices using both qualitative and quantitative research methods;  (2) develop and test an effective smoking cessation intervention for the Syrian environment; and  (3) train Syrian researchers. | 1.Household survey 2. epidemiology surveys 3.in depth ethnographic interviews | 18–65 years | males & females | Syria | cigarette, water pipe smoking | Routine and  occasional |
| Ayub et al 2015 | Jordan tobacco dependence treatment guidelines: rationale and development | 1. To compile all information related to tobacco dependence treatment that is relevant to Jordan in a single resource  2. to review the international research and guidelines on tobacco dependence treatment and create a comprehensive, standardized, evidence-based, yet locally customized reference; 3. to support ongoing educational efforts in the country; 4. to reinforce the message that a systematic means of tobacco dependence treatment is required for implementation within health care settings; 5. to provide an officially endorsed guideline to assist decision-makers responding to the call for integration of tobacco dependence treatment services into their systems; and 6. to reaffirm the Jordanian MoH’s commitment to the World Health Organization’s framework Convention on Tobacco Control (WHO FCTC) | 1. Formation of the Jordan Tobacco Dependence Treatment Guidelines Group.  2. national situation analysis. 3.Outline and content development  4. National review  5. Endorsement  and launch. | NR | NR | Jordan | NR | NR |
| Uang et al 2017 | Smokefree implementation in Colombia: Monitoring, outside funding, and business support | To analyse successful national smokefree policy implementation in Colombia, a middle-income country. | Key informant interviews | NR | NR | Colombia | Waterpipe, cigarettes | NR |
| Bader et al 2017 | Informing tobacco control policy in Jordan: assessing the effectiveness of pictorial warning labels on cigarette packs | To assess the effectiveness of the new set of PWLs after having them circulate on the market for 2.5 years. | Cross-sectional | 17–26 years | males & females | Jordan | smoke (cigarettes) | NR |
| Odukoya et al 2016 | Tobacco Cessation Interventions in Tertiary Hospitals in Nigeria: An Audit of Patient Records | To assess brief intervention tobacco cessation activities documented by physicians in some select tertiary hospitals in Nigeria. | cross-sectional descriptive study was carried out using information abstracted from patients’ health records  (Retrospective record-based study) | 12 years and older | Males & Females | Nigeria | Smoked tobacco, smokeless tobacco, cigarettes, cigars, regular pipes, water pipes/ shisha, tobacco. | NR |
| Asare et al 2019 | Effects of Prices on Youth Cigarette Smoking and Tobacco Use Initiation in Ghana and Nigeria | Estimating the effect of cigarette prices on youth smoking and tobacco use initiation in Nigeria and Ghana. | Secondary data analysis with Global Youth Tobacco Survey (GYTS) being main data source | 11–18 years | Males | Ghana, Nigeria | cigarette | Never users and routine users |
| Maldonado 2022 | Tobacco Taxes as the Unsung Hero: Impact of a Tax Increase on Advancing Sustainable Development in Colombia | To evaluate the expected effects of a tobacco tax increase on the Sustainable Development Goals in Colombia. | The artificial society and the static microsimulation was built in three consecutive steps: 1) construction of the synthetic dataset, 2) simulation of behaviours and states, and 3) estimation of aggregate indicators from the simulated microdata. | 10-64 years | Males & Females | Colombia | cigarette | not clear |
| Andreeva et al 2011 | Recall of tobacco pack health warnings by the population in Ukraine and its association with the perceived tobacco health hazard | To estimate potential contribution of THW to smoking decline process in Ukraine. | Nationwide omnibus survey | 18 and above | Males & Females | Ukraine | Smoke | current smokers (daily, occasional and reducers), former smokers (former daily and former occasional smokers) and non-smokers (experimenters and never smokers) |
| Egbe 2018 | Role of stakeholders in Nigeria's tobacco control journey after the FCTC: lessons for tobacco control advocacy in low-income and middle-income countries | To assess the tobacco industry’s strategies to oppose tobacco control and strategies used by tobacco control advocates to push for FCTC-compliant legislation in Nigeria | Qualitative study- key informant interviews (news, articles and written materials were also used) | NR | NR | Five local and international organisations involved in tobacco control advocacy in Nigeria. | NR | NR |
| Adebiyi 2016 | Perceived effectiveness of graphic health warnings as a deterrent for smoking initiation among adolescents in selected schools in southwest Nigeria | To evaluate the perceived effectiveness of selected graphic warnings on smoking initiation amongst in-school adolescents. | Cross-sectional study | secondary school students 13-17 years | Males & Females | School, Igbo-Ora, Nigeria | cigarette smoking | not specified |
| Perl et al 2014 | Responses to antismoking radio and television advertisements among adult smokers and non-smokers across Africa: message-testing results from Senegal, Nigeria and Kenya | To examine whether adaptation of existing antitobacco television and radio advertisements (ads) from high-income countries is a viable tobacco control strategy for Africa | 14-item quantitative rating scale, structured group discussions led by a moderator. | Male and female adult smokers (smoked daily in the past [year) and non-smokers (<100 cigarettes in their lifetime and did not currently smoke) aged 18–40 years | Males & Females | Urban setting in Senegal, Nigeria, and Kenya | cigarette smoking | smokers (smoked daily in the past year) and non-smokers (<100 cigarettes in their lifetime and did not currently smoke) |
| Hussain et al 2018 | School-based behavioural intervention to reduce the habit of smokeless tobacco and betel quid use in high-risk youth in Karachi: A randomized controlled trial | To develop (a) a BCI that was pertinent to the sociocultural aspects of South Asian SLT users, followed by (b) assessing its efficacy in changing knowledge and perceptions regarding SLT use and cessation in a high-risk group of 11–16-year-old school-going children in Karachi who attended both private and public-sector schools. | Randomized controlled trial | 11–16 years | Males & Females | 26 public and private sector schools Karachi, Pakistan | smokeless tobacco and betel quid | not specified |
| Hallit et al 2019 | The impact of textual and pictorial warnings on tumbac (waterpipe tobacco) boxes on the motivation and intention to quit waterpipe smoking in Lebanon: a cross-sectional study | To assess the impact of textual vs. pictorial warnings on tumbac boxes, on the motivation to quit and the intention to quit among Lebanese waterpipe smokers. | Cross-sectional study | 18 years of age or above | Males & Females | Lebanon | tumbac (waterpipe tobacco) | current (≥ 1 waterpipe per week) and exclusive waterpipe smokers |
| Mapa-Tassou 2018 | Two decades of tobacco use prevention and control policies in Cameroon: results from the analysis of non-communicable disease prevention policies in Africa | Explores the extent to which tobacco use and prevention policies in Cameroon align with the WHO tobacco “best buy” interventions. Secondly, it describes the context, the content, the formulation and implementation process of these policies and their effectiveness. | Qualitative case study design | NR | NR | Africa | NR | NR |
| Girvalaki et al 2020 | Impact of the ENSP eLearning platform on improving knowledge, attitudes, and self-efficacy for treating tobacco dependence: An assessment across 15 European countries | To evaluate the effectiveness of ENSP eLearning curriculum in increasing healthcare professionals’ knowledge, attitudes, self-efficacy (perceived behavioural control) and intentions in delivering tobacco treatment interventions in their daily clinical routines. | Quasi-experimental pre-post design | <30 to ≥70 | Males & Females | Albania, Armenia, Belgium, Italy, France, Georgia, Greece, Kosovo, Romania, North Macedonia, Russia, Serbia, Slovenia, Spain, Ukraine | Tobacco (Smoke) | Smoker, Ex-smoker, Non-smoker |
| Chopra et al 2014 | Communicating tobacco health risks: How effective are the warning labels on tobacco products? | To investigate the awareness and effectiveness of warning labels on tobacco products among health and non-healthcare professional of Barwala, Panchkula. | Descriptive cross-sectional survey | NR | Males & Females | Haryana, India | NR | Current tobacco users, non-tobacco users, past tobacco users |
| Hnin et al 2020 | Awareness and Perceptions on Health Warning Labels on Cigarette Packs among Smokers: A Cross-Sectional Study | Examining the awareness and perceptions of HWLs on cigarette packs among smokers. | Cross-sectional survey | 18 and older | Males & Females | Mandalay, Myanmar | Cigarette (Smoke) | smoked at least 100 cigarettes in their lifetimes (Everusers) |
| [Creating a smoke-free city – Balanga City, the Philippines](https://www.who.int/news-room/feature-stories/detail/creating-a-smoke-free-city-balanga-city-the-philippines) | | To create a smoke-free Balanga city | NR | NR | NR | Philippines | Not specified | NR |
| [Johnson & Johnson donates nicotine patches to help thousands of Jordanians quit tobacco during COVID-19](https://www.who.int/news-room/feature-stories/detail/Johnson-Johnson-donates-nicotine-patches-to-help-thousands-of-jordanians-quit-tobacco-during-COVID-19) | | To help people quit tobacco during COVID-19 pandemic | NR | NR | NR | Jordan | Not specified | NR |
| [WHO urges more countries to require large, graphic health warnings on tobacco packaging](https://www.who.int/news/item/09-08-2011-who-urges-more-countries-to-require-large-graphic-health-warnings-on-tobacco-packaging) | | To require large, graphic health warnings on tobacco packaging | NR | NR | NR | Multiple | NR | NR |
| [World governments show unity against tobacco industry and agree to new tobacco-control measures](https://www.who.int/news/item/10-12-2010-world-governments-show-unity-against-tobacco-industry-and-agree-to-new-tobacco-control-measures) | | Parties to the World Health Organization Framework Convention on Tobacco Control (WHO FCTC) unanimously adopted a number of decisions this week strengthening tobacco-control efforts worldwide.  The fourth session of the Conference of the Parties (COP4) comes as a number of countries are facing growing pressure from the tobacco industry.  In response, Parties to the Convention adopted a Declaration proposed by the host country, Uruguay, reaffirming their strong commitment to prioritize health measures and to exchange information on the industry’s activities which attempt to interfere with the implementation of public health policies. | NR | NR | NR | Multiple | NR | NR |
| [WHO report finds dramatic increase in life-saving tobacco control policies in last decade](https://www.who.int/news/item/19-07-2017-who-report-finds-dramatic-increase-in-life-saving-tobacco-control-policies-in-last-decade) | | NR | NR | 11-17 | Males & Females | Multiple | NR | NR |
| [CATCH approach for smokeless tobacco cessation in the South-East Asia region](https://www.who.int/publications/i/item/catch-approach-for-smokeless-tobacco-cessation-in-the-south-east-asia-region) | | to understand the needs and challenges of SLT cessation in five identified countries of the SEAR–Bangladesh, Bhutan, India, Myanmar and Nepal–that have high prevalence of SLT consumption among adults and youth. The document strives to identify opportunities for taking this public health agenda forward in these five countries and to lay down specific priorities for action and intervention. | NR | NR | NR | SEA region | Smokeless | NR |
| [Hiding Tobacco Products at Convenience Stores Reduces Teens' Risk of Future Tobacco Use](https://www.rand.org/news/press/2015/11/23.html) | | To examine whether limiting displays of cigarettes and other tobacco products in retail outlets can reduce the intention of young people to begin smoking. | NR | NR | NR | US | NR | NR |
| [Graphic Warning Labels on Tobacco Packages Can Deter Some Smokers from Buying Cigarettes](https://www.rand.org/news/press/2019/04/09.html) | | the study found that the effects of graphic health warning labels on adult smokers' purchase of cigarettes depend on their level of nicotine dependence. | NR | NR | NR | US | NR | NR |
| [Reducing Teen Smoking and Drinking through Increased Schooling in the Dominican Republic](https://www.povertyactionlab.org/evaluation/reducing-teen-smoking-and-drinking-through-increased-schooling-dominican-republic) | | To examine the effect of increased schooling and decreased work on smoking and drinking behavior among secondary school-aged boys. | NR | NR | NR | Dominican Republic | NR | NR |

**Supplementary table 4: Details of intervention**

| **Study ID** | **Name of the intervention** | **Type of intervention** | **Stakeholders engaged** | **Mode of intervention delivery (peer led/health worker led)** | **Details of the intervention provider (For each category of intervention provider (e.g. psychologist, nursing assistant)** | **Was technology used in delivering this intervention (Yes/ No)** | **What materials were used to deliver the intervention?** | **Procedures used in the intervention (: Describe each of the procedures, activities, and/or processes used in the intervention** |
| --- | --- | --- | --- | --- | --- | --- | --- | --- |
| Ward et al 2006 | Tobacco cessation interventions | one-one | Medical anthropologists, psychologists, epidemiologists, behavioural pharmacologists, and physicians | Health worker led | clinic-wide strategies to identify smokers and assist them in quitting (for example, identification of all patients’ smoking status during intake assessment, providing training to all primary care physicians to deliver a brief cessation intervention to all smokers, and delivering intensive cessation intervention aided by NRT). | Yes | Interative learning/application was applied.  Distance learning and mentorship was implemented.  Syrian researchers related to expert mentors in their field of interest for a year-long training course.  Three Syrian researchers underwent this type of training with a focus on cessation intervention delivery.  This training covered course materials based on Fiore et al28 involving a combination of self-readings, homework, report writing, and quizzes. | “5A” approach (ask about smoking status, advise about the importance of quitting, assess willingness to quit, assist by providing basic cessation guidelines, and arrange for follow-up assistance). 50 smokers to either a brief (single session) or intensive (four face-to-face sessions plus six phone follow-ups) hospital-based, free, behavioural counselling intervention. |
| Ayub et al 2015 | Policy intervention, Development of guidelines | NR | King Hussein Cancer Centre (KHCC) and Jordanian MoH | NR | NR | No | NR | NR |
| Uang et al 2017 | NR | population based | Ministerio de Salud y Protección Social (Health Ministry”)  Instituto Nacional de Cancerología (National Cancer Institute)  Procuraduría General de la Nación (National Inspector General)  Local health departments  Local police  Liga Colombiana Contra el Cáncer (Colombian League Against Cancer)  Corporate Accountability International  Fundación para la Educación y el Desarollo Social FES (Social equity organization)  Asociación de Bares de Colombia (Asobares; Bar Owners’ Association of Colombia)  Compañía Colombiana de Tabaco ( Colombian Tobacco Company, Coltabaco)  Productora Colombiana de Tabaco (Colombian Tobacco Producer, Protabaco)  Federación Nacional de Comerciantes (Fenalco) | Smoke free Education, trainings and monitoring of local health department staff  Enforcement by local police  Colombia’s positive smokefree messaging (signage) to improve compliance  Education to bar owners for 6 months. | NR | No | Signage for health education candy for positive reinforcement | Indoor smokefree-Enclosed areas of workplaces and public places (including bars, restaurants, pubs, casinos, nightclubs)   Entirety smokefree, including outdoors- Health establishments Education/museums/libraries Sports/cultural spaces Places for youth Places for industrial activity transportation for the public (including taxis)  Signage- Required, without specific message text  Fines- Starting at 1 monthly minimum wage (in 2009 was 496 900 Colombian pesos29or about 250 dollars) or suspension of health license  Indoor smokefree-Enclosed areas of workplaces and public places (including bars, restaurants, pubs, casinos, nightclubs)  Entirety smokefree, including outdoors- Health establishments Education/museums/libraries Sports/cultural spaces Places for youth Places for industrial activity transportation for the public (including taxis)  Signage- Required, without specific message text  Fines- Starting at 1 monthly minimum wage (in 2009 was 496 900 Colombian pesos29or about 250 dollars) or suspension of health license  education for bar owners for six months including provisions of brochures, and bar coasters. Also provided candy to people who stepped outside to smoke, had models come to bars to give prizes to such people, and worked with the Bogotá government on a protocol for the emergency services number (123), which would summon police to eject patrons who insisted on smoking indoors. |
| Bader et al 2017 | Pictorial warning labels (PWLs) | population based | Volunteers from four university campuses (Jordan University of Science and Technology in the northern region, University of Jordan and Hashemite University in the central region, and Mutah University in the southern region)  surveyors from the International Federation of Medical Students’ Associations - Jordan Chapter | health worker (medical students) | 25 volunteers were recruited through Jordan’s chapter of the International Federation of Medical Students Association to serve as surveyors | NR | NR | Respondents notice and recall of the PWLs in circulation and their general reactions to these PWLs |
| Odukoya et al 2016 | Tobacco Cessation Interventions in tertiary care hospitals | Population based | Patient data was retrospectively used | Health worker led (retrospective) | NR | NR | No intervention was provided during study (retrospective) | Tobacco Cessation Interventions were documented. The documentation of tobacco use and the pattern of tobacco cessation services provided to identified tobacco users, using the 5A’s of tobacco cessation as a guide (Physician offered some advice, Physician assessed patients’ readiness to quit, Physician assisted patient with quitting, Type of assistance offered (Counselling, medication, set a quit date, Physician referred/Arranged for follow-up) |
| Asare et al 2019 | Increase in cigarette prices | Population based | Not applicable | NR | NR | NR | NR | Increase in Prices on Cigarette and Tobacco |
| Maldonado 2022 | Tobacco tax increase (simulation) | Population based | NR | Higher tobacco taxes in advancing Sustainable Development in Colombia. | Sustainable Development goals initiative | No | Simulation | A tax hike in the specific component of the excise tax from COP$2,350 to COP$7,000 |
| Andreeva et al 2011 | Recall of tobacco pack health warnings by the population in Ukraine | Population based | Nationwide survey by Kyiv International Institute of Sociology (KIIS) | No intervention was provided in the study | NR | NA | No intervention was provided in the study | tTo measure the respondent’s attention to health warnings, study participants were asked to describe the warnings they recall. Interviewers were instructed not to show the texts to the respondents and not to expect the exact wording of the warning, but to mark the warning as ‘recalled’ given the respondent mentions the corresponding topic (for instance ‘impotence’, ‘heart disease’, ‘pregnancy’ etc.). Number of the warnings that were recalled was also used as a measure of the respondent’s attention to health warnings with minimum number of health warnings recalled equal to 0, and maximum equal to 7. |
| Egbe 2018 | NR | One on one interviews were conducted | Organisations involved in tobacco control  Tobacco industry third party allies and front groups  National organisations (perceived to be protective of the tobacco industry) | NR | NR | NR | NR | Both tobacco control advocates and industry used discursive (argument-based) and instrumental (activity-based) strategies |
| Adebiyi 2016 | Graphic health warnings as a deterrent for smoking initiation among adolescents | Population based | A research assistant was involved | health worker led | Medical students, research assistant | Yes | Digital projection | The graphic images were introduced one at a time by digital projection with an interval of 5 min apart before students were asked to record their responses in the questionnaire provided. The images included pictures of Cigarette smoking harms children, cigarette smoking causes cancer of the airways, cigarette smoking causes stroke, Cigarette smoking causes impotence. Four images; Figs. 1, 2, 3, and 4 (from Thailand, Taiwan, Mauritius, and France respectively) were obtained from the pictorial health warning galleries of the World Health Organization and were shown to the respondents’ one after the other |
| Perl et al 2014 | Antismoking radio and television advertisements | Population based | Collaboration with government officials and partners in Africa | Five TV ads and five radio ads | Government officials and partners in Africa | Yes | Television and radio | 5 radio ads that included: Emphysema Sufferer Keith, Industry Spokesman, Coughing Child, suffering, bronchoscopy. Tv ads includes Cigarettes are Eating your baby alive, Break the Chain, lung, sponge & industry revealed. Five TV ads and five radio ads were chosen in close collaboration with government officials and partners in Africa. Advisers selected ads nominated by partners in Africa and found in World Lung Foundation’s Mass Media Resource (worldlungfoundation.org/mmr), a repository created by leading experts. Criteria included strong performance in ads’ countries of origin, African stakeholders’ assessment of their effectiveness and ease of adaptation. |
| Hussain et al 2018 | Behaviour changing intervention (BCI) | Group (school clusters) | NR | NR | MSc. DS students (Oral Pathology) | No | Not clear | The methodology acquired in designing, developing, and assessing the accuracy of this BCI was based on the Medical Research Council framework for complex interventions. The components of BCI included: Identifying the SLT and BQ products, their harmful effects, and why is it important to quit along with setting a quit date • Preparing to quit along with managing urges for relapse, and • Recognizing and managing withdrawal symptoms |
| Hallit et al 2019 | Impact of textual and pictorial warnings on tumbac (waterpipe tobacco) boxes | Population based | tobacco control organizations, ministries of health, academic institutions, and nongovernmental organizations | health worker led | Research assistants | No | a text warning on the front and back of the packaging | textual and pictorial warnings on tumbac (waterpipe tobacco) boxes.  rules and regulations were implemented on tobacco products require a text warning on the front and back of the packaging, and in 2011, the legislation expanded the minimum requirement for those warnings to 40% of the packaging surface area |
| Mapa-Tassou 2018 | Tobacco use prevention and control policies in Cameroon | Population based | Government and policy makers | NR | NR | NR | NR | A structured review of 19 government policy documents related to tobacco use and prevention, in-depth interviews with 38 key stakeholders and field observations.  The Walt and Gilson’s policy analysis triangle was used to describe and interpret the context, content, processes and actors during the formulation and implementation of tobacco prevention and control policies. |
| Girvalaki et al 2020 | ENSP eLearning platform | Population based | EPACTT-Plus, collaborating institutions from 15 countries (Albania, Armenia, Belgium, Italy, France, Georgia, Greece, Kosovo, Romania, North Macedonia, Russia, Serbia, Slovenia, Spain, Ukraine) worked to develop an accredited eLearning course on Tobacco-Treatment Delivery | health worker led | European Network for Smoking Cessation and Prevention (ENSP) | Yes | An eLearning curriculum was developed to train healthcare professionals in the guideline recommendations, available in 14 languages. | Five modules were included. Module 1: This module is entitled ‘Nicotine Addiction - Why people smoke?  Module 2: This module is entitled ‘How to help your patients quit smoking’.  Modules 3 and 4: The behavioural counselling content is divided into two parts, Modules 3 and 4.  In Module 3, the role of behavioral counselling as a treatment for supporting tobacco users with quitting is discussed as well as evidence-based counselling strategies designed for individuals ready to quit smoking in order to increase their likelihood of successfully quitting. In Module 4, key behavioral strategies used to enhance motivation among patients who are not ready to quit smoking include motivational interviewing and smoking reduction approaches. Module 5: This module provides training on pharmacological treatment as a fundamental component of treating tobacco use. The curriculum includes five different training modules as well as case studies, quizzes, and interactive content. The course was developed in Moodle. At the end of each module, a knowledge assessment is completed by users.  In 2018, the European Network for Smoking Cessation and Prevention (ENSP) released an update to its tobacco treatment guidelines for healthcare professionals5. The guidelines summarize the latest evidence and provide recommendations for healthcare professionals. An eLearning curriculum was developed to train healthcare professionals in the guideline recommendations, available in 14 languages. |
| Chopra et al 2014 | Warning labels on tobacco products | Population based | NR | NR | The Cigarettes and Other Tobacco Products (COTPA) (Prohibition of Advertisement & Regulation of Trade & Commerce, Production, and Supply & Distribution) Act, 2003 in India | No | Pictorial warning label | Pictorial warnings (A picture of lung disease and oral cancer) were shown to the health care and non-health care professionals. |
| Hnin et al 2020 | Health Warning Labels on Cigarette Packs | Population based | Myanmar approved legislation for HWLs (Health warning labels) on cigarette packs, and the law came into effect on 1 September 2016. The local authorities and basic health staff were involved in data collection | NR | NR | No | Images | Ten images with corresponding text messages are ordered for printing on packages. Each HWL is used for one year, and it will be rotated to the next one in the series. This will be also applied to local products (cheroot packages). It is required to cover 75% of the front and back surfaces of tobacco product packing, in which the image is to cover 50% and the text message is to cover 25%. |
| [Creating a smoke-free city – Balanga City, the Philippines](https://www.who.int/news-room/feature-stories/detail/creating-a-smoke-free-city-balanga-city-the-philippines) | Ban on tobacco products in all public places and public utility vehicles in the city’s University Town area. | Population based | Policymakers | NR | NR | NR | Policy | in 2010 the city council passed a Comprehensive No-Smoking Ordinance and declared 31 May as “The City of Balanga’s No Tobacco Day”. The city also initiated a “Tobacco-Free Generation” campaign in order to eliminate tobacco smoking among those born after 2000.  In 2013, Mayor Garcia then wrote to neighboring municipalities to request a halt to “blitzkrieg promotional activities” conducted near city borders by the tobacco industry. In 2014, the city launched a campaign to promote healthy lifestyles during adolescence, including not smoking tobacco. And for the 2015 World No Smoking Month, the city launched a campaign entitled “I love my family, Say no to Cigarettes” |
| [Johnson & Johnson donates nicotine patches to help thousands of Jordanians quit tobacco during COVID-19](https://www.who.int/news-room/feature-stories/detail/Johnson-Johnson-donates-nicotine-patches-to-help-thousands-of-jordanians-quit-tobacco-during-COVID-19) | Nicotine patches | Population based | NR | NR | NR | NR | NR | The Ministry of Health of Jordan has received its first ever donation of nicotine replacement therapy from Johnson & Johnson Consumer Health through its partnership with the Access Initiative for Quitting Tobacco (AIQT). The donation, at an estimated retail value of about US$ 800,000, aims to support thousands of Jordanians and refugees quit smoking during the COVID-19 pandemic and beyond. |
| [WHO urges more countries to require large, graphic health warnings on tobacco packaging](https://www.who.int/news/item/09-08-2011-who-urges-more-countries-to-require-large-graphic-health-warnings-on-tobacco-packaging) | Graphic health warnings | Population based | NR | NR | NR | NR | NR | More than one billion people in 19 countries are now covered by laws requiring large, graphic health warnings on packages of tobacco, nearly double the number of two years ago, when only about 547 million people were covered in 16 countries, WHO reports today in its third periodic report on the global tobacco epidemic. |
| [World governments show unity against tobacco industry and agree to new tobacco-control measures](https://www.who.int/news/item/10-12-2010-world-governments-show-unity-against-tobacco-industry-and-agree-to-new-tobacco-control-measures) | Strengthening tobacco-control efforts worldwide | Population based | NR | NR | NR | NR | NR | The Conference decided that:  flavoring ingredients that increase attractiveness of tobacco products should be regulated in order to reduce the number of new smokers, especially among youth; smoking cessation services should be integrated into national health systems to make them more available for increasing number of smokers who wish to quit; and parties should establish an infrastructure and build capacity to support education, communication and training, thereby raising public awareness and promoting social change. |
| [WHO report finds dramatic increase in life-saving tobacco control policies in last decade](https://www.who.int/news/item/19-07-2017-who-report-finds-dramatic-increase-in-life-saving-tobacco-control-policies-in-last-decade) | Tobacco control policies (MPOWER) | Population based | NR | NR | NR | NR | NR | NR |
| [CATCH approach for smokeless tobacco cessation in the South-East Asia region](https://www.who.int/publications/i/item/catch-approach-for-smokeless-tobacco-cessation-in-the-south-east-asia-region) | CATCH approach | Population based | NR | NR | NR | NR | NR | NR |
| [Hiding Tobacco Products at Convenience Stores Reduces Teens' Risk of Future Tobacco Use](https://www.rand.org/news/press/2015/11/23.html) | Hiding Tobacco Products at Convenience Stores Reduces Teens' Risk of Future Tobacco Use | Population based | NR | NR | NR | NR | NR | NR |
| [Graphic Warning Labels on Tobacco Packages Can Deter Some Smokers from Buying Cigarettes](https://www.rand.org/news/press/2019/04/09.html) | Graphic health warnings | Population based | NR | NR | NR | NR | NR | NR |
| [Reducing Teen Smoking and Drinking through Increased Schooling in the Dominican Republic](https://www.povertyactionlab.org/evaluation/reducing-teen-smoking-and-drinking-through-increased-schooling-dominican-republic) | Increased schooling | Population based | NR | NR | NR | NR | NR | NR |

**Supplementary table 5: Details of intervention and outcomes**

| **Study ID** | **Where was the intervention delivered? (Describe the type(s) of location(s) where the intervention occurred, including any necessary infrastructure or relevant features.)** | **Duration of intervention delivery (Expressed in days, months, years; Describe the number of times the intervention was delivered and over what period, including**  **the number of sessions, their schedule, and their duration, intensity, or dose. )"** | **Intervention tailoring (If the intervention was planned to be personalised, titrated, or adapted, then describe what, why,**  **when, and how** | **Modifications done to the interventions (If the intervention was planned to be personalised, titrated, or adapted, then describe what, why,**  **when, and how** | **Outcomes of intervention** | **Challenges faced during the delivery of these interventions** |
| --- | --- | --- | --- | --- | --- | --- |
| Ward et al 2006 | Primary care centres and one private diabetes clinic. | A brief (single session) or intensive (four face-to-face sessions plus six phone follow-ups) hospital-based, free, behavioural counselling intervention. | Personalized and phone follow ups were used | NR | The prevalence of cigarette smoking was 56.9% among men and 17.0% among women, while the prevalence of waterpipe smoking was 20.2% among men and 4.8% among women.  Daily use predominated for cigarettes (29.0%), while the opposite was seen in waterpipe use with 10.6% smoking occasionally. Interest in quitting was greater for cigarette than waterpipe smokers (74.0% v 48.6%), while quit rates were higher for waterpipe compared to cigarettes (28.2% v 16.5%). | NR |
| Ayub et al 2015 | NR | NR | NR | NR | Development of Jordan’s tobacco dependence treatment guidelines, the first comprehensive Arabic-language tobacco dependence treatment guidelines developed in the region, drafted and reviewed by a team of local and international experts, and endorsed by Jordan’s national health authority. | NR |
| Uang et al 2017 | Across Colombia. | NR | NR | NR | Colombia’s Ministry of Health coordinated local implementation practices.  Nongovernmental organizations provided technical assistance and highlighted noncompliance.  The bar owners’ association provided concerted education campaigns.  Tobacco interests did not openly challenge implementation.  Health organization monitoring, external funding, and hospitality industry support contributed to effective implementationColombia’s Ministry of Health coordinated local implementation practices. Implementation was strongest in big cities and in cities with supportive political leadership: Bogotá (population 8 million), Medellín (2.4 million), Cali (2.3 million), Colombia’s most influential cities, and two southwestern cities, Popayán (250 000) and Pasto (480 000), with personally committed mayors.  Implementation was weakest in rural areas and the Atlantic coast, with less interest from agencies in these areas.  Nongovernmental organizations provided technical assistance and highlighted noncompliance.  The bar owners’ association provided concerted education campaigns.  Tobacco interests did not openly challenge implementation.   Health organization monitoring, external funding, and hospitality industry support contributed to effective implementation | representatives friendly to the tobacco industry opposed it  Tobacco companies and third-party allies- Opposed adoption but did not openly oppose implementation |
| Bader et al 2017 | Four university campuses (Jordan University of Science and Technology in the northern region, University of Jordan and Hashemite University in the central region, and Mutah University in the southern region. Respondents from the various schools on his/her own university campus were selected. | Effectiveness surveyed in 2015 to perceptions gauged in 2010 during a pre-launch evaluation exercise. | NR | NR | Among smokers, 63.1% (195/309) reported seeing PWLs frequently (every time or some of the times they held a cigarette packet). Of those, 36.4% (71/195, p < 0.05) reported being influenced by the PWLs enough to consider quitting and 31.8% (62/195) reported avoiding looking at them. Among smokers recalling at least one of the statements associated with PWLs on cigarette packs, 32.2% (66/205, p < 0.05) reported avoiding looking at them. A significant majority (66.9%, 113/169, p < 0.0001) also reported PWL4 to motivate quitting. | NR |
| Odukoya et al 2016 | Six teaching hospitals in three of the six geopolitical zones of the country | last 6 months prior to the study | NR | NR | Among identified tobacco users, it was documented that 12.9% were offered some form of tobacco cessation advice; readiness to quit was recorded in 2.6%; assistance with quitting was documented for 1.5% of tobacco users, while only 0.8% showed documentation of patient follow-up. | NR |
| Asare et al 2019 | Nigeria and Ghana population | From 2018 intervention was introduced | High taxes were applied to the countries such as Nigeria, etc., include first-year students of senior secondary schools | NR | Effects of Cigarette Prices on Current Cigarette Smoking: In the two-country analysis, a 1% increase in cigarette prices decreased cigarette smoking by 0.9%. Effects of Cigarette Prices on Tobacco Use Initiation: Students in Ghana accumulated more than 40% risk of initiation before age 16. Students in Nigeria faced a lower accumulated risk at age 16 compared to Ghana. | NR |
| Maldonado 2022 | NR | NR | NR | NR | The tobacco tax hike reduces the number of smokers (from 4.51 to 3.45 MM smokers) and smoking intensity, resulting in a drop in the number of cigarettes smoked in Colombia (from 332.3 to 215.5 MM of 20-stick packs). | NR |
| Andreeva et al 2011 | Ukraine | 2006, 2008, 2009 | Intervention was provided to the population of smokers | NR | quitting smoking after 2006 and perception of tobacco-related 2 hazards. Higher odds for smokers to recall a health warning compared to non-smokers were more prominent (OR 3,37 (95% CI 2,56-4,42)) for the front-side warning and the weakest for the warnings related to passive smoking ('Protect children: do not make them inhale your smoke' – OR 1,23 (95% CI 0,96-1,57), and 'Tobacco smoke harms the health of those around you' – OR 1,35 (95% CI 1,09-1,67). | NR |
| Egbe 2018 | NR | December 2015 and June 2017 | NR | NR | Framework Convention on Tobacco Control (FCTC) ratification has not stopped the tobacco industry from using wellestablished tactics to stall tobacco control policy in Nigeria. The Policy Dystopia Model and WHO categories of tobacco industry interference provide a helpful framework for analysing and understanding the activities of the tobacco industry and of tobacco control advocates in Nigeria. Tobacco control advocates in Nigeria were assisted through international technical support and funding. Efforts of Nigeria’s tobacco control advocates helped in enacting a law which partially implemented the FCTC. These lessons from Nigeria are transferable and adaptable for other low-income and middle-income countries and African countries | the Policy Dystopia Model and WHO categories presented challenges relating to the categorisation of argument and activity-based strategies and within WHO strategies of interference due to blurred differentiating lines between some strategies, for example, ‘manipulating public opinions’ was chiefly carried out by the industry under the guise of CSR.  Attempts to reach government officials from the National Assembly and the Federal Ministry of Health for interviews were unsuccessful, making it impossible to verify some claims by the key informants. Advocates working in Nigeria were also sometimes difficult to reach leading to delays in the data collection. Many of the advocates who previously worked for tobacco control in Nigeria have retired or are no longer engaged in tobacco control. These advocates could not be reached to provide information about early tobacco control advocacy activities in Nigeria. |
| Adebiyi 2016 | Selected schools classrooms | NR | NR | No | Pictorial health warnings that evoke fear may likely influence the decision to initiate smoking of cigarettes by adolescents. With all the images, fear was the dominant emotion expressed by the respondents. This was expressed by 307 (56.4), 215 (39.5), 203 (37.3) and 228 (41.9 %) respondents to images 1, 2, 3, and 4 respectively. Furthermore, 76.7, 44.7, 58.5 and 62.1 % of respondents felt Images 1, 2, 3 and 4 respectively will to a large extent prevent people from initiating smoking. There was no association between perceived effectiveness and gender. However, those younger than 15 years rated images on cancer of the airway and impotence as probably effective to a larger extent than did those who were 15 years and older (p = 0.032). | NR |
| Perl et al 2014 | Radio & tv ads | Not described | NR | Yes (the ad was unfairly targeting black people and negatively characterising them—the anti-industry television ad was always shown last to avoid potential ‘contamination’ of the others). | Significant main effects of television ads were found for all measures (all p<0.001) other than Quitting Preparedness, where a trend towards an effect was found (p=0.060). Higher ratings on PE were given by non-smokers, older participants, women, those with children and those without tertiary degrees (all at least p<0.05). Higher Anti-Industry ratings were also given by those in smaller urban locations and in homes where smoking was permitted indoors (all at least p<0.05). Lower ratings on Commitment to Avoiding Tobacco were given by younger participants and males (all p<0.05).  Perceived Effectiveness (PE), Anti-industry/government support, Commitment to avoiding tobacco (non-smokers only), Quitting Preparedness (smokers only), Single-item outcome | owing to cost considerations, only basic ad adaptations were tested, which could have interfered with their performance. However, the successful performance of the ads, despite minimal adaptation, suggests that they may perform more strongly with appropriate contextualisation. |
| Hussain et al 2018 | In schools | The first intervention was conducted during the period of April 25 –May 07, 2016, with simultaneous visits to the control school clusters.  For the intervention clusters, follow-up visits were conducted after 2 weeks (for the second intervention) during the period of May 09–18, 2016 Second follow-up visits for both the intervention and control schools (12 weeks after the first intervention) were done during the period of July 30, 2016 –Aug 12, 2016 | NR | NR | In the intervention arm, the starting rate was 44.7% and was reduced to 11.6%. The knowledge related to the transformation of oral white patch/lesion into oral cancer increased from 78% to 97.2% in intervention group. . The perception regarding its harmful effect on their health changed/improved to 91.3% from 78.3% in the intervention group. | NR |
| Hallit et al 2019 | 25 selected cafes and restaurants from three out of five districts of Lebanon | January and May 2018 | NR | No data | Having a high motivation (ORa = 2.61), thinking that using shocking images on tumbac boxes can have more effect than textual warnings (ORa = 2.12), those who stopped smoking because of the warnings (ORa = 2.62), those who would choose pictorial warnings alone (ORa = 2.11), and both pictorial and textual warnings (ORa = 3.41) on tumbac packages were associated with higher intention to quit WS in two months. | NR |
| Mapa-Tassou 2018 | NR | Documents review was done between mid-2014 to the end of 2015 | First, it explores the extent to which tobacco use and prevention policies in Cameroon align with the WHO tobacco “best buy” interventions. Secondly, it describes the context, the content, the formulation and implementation process of these policies and their effectiveness. | NR | Twelve out of 19 policies for tobacco use and prevention address the WHO “best buy” interventions. Cameroon policy formulation was driven locally by the social context of non-communicable diseases, and globally by the adoption of the WHO Framework Convention on Tobacco Control. | NR |
| Girvalaki et al 2020 | 15 countries (Albania, Armenia,Belgium, Italy, France, Georgia, Greece, Kosovo, Romania, North Macedonia, Russia, Serbia, Slovenia, Spain, Ukraine) | Pre-test and post-test were followed ((Dec 2018-Juy 2019) | NR | No data | Significant improvements were documented in intentions to address tobacco use as a priority, document tobacco use, offer support, provide brief counselling, give written material, discuss available medication, prescribe medication, schedule dedicated appointment to develop a quit plan, and be persistent in addressing tobacco use with the patients (all p<0.001). | However, as the eLearning course was accredited, there were some restrictions on the procedure of the completion of the course, as participants had to successfully complete each module’s test to receive accreditation. The course healthcare professionals may have over-performed in their responses for changing attitude norms, perceived behavioral control and intentions in delivering tobacco treatment interventions with their patients who smoke. |
| Chopra et al 2014 | Barwala, Panchkula, Haryana, India (Colleges-nursing, dentistry, pharmacy, engineering) | April to May 2013 | NR | NR | 19.1% health professionals and 26.7% non-health professionals were current smokers. Healthcare professionals were more aware about harmful effects of tobacco as compared to non-healthcare professionals (P-value < 0.05). More that 70% believe that warnings create awareness about health hazards of tobacco and help in reducing or quitting tobacco. Pictorial warning was found to be better as compared to text warning. A picture of lung disease and oral cancer was correctly identified by >70% health professionals and >40% by non-health professionals. Most effective warning label, the picture of oral cancer (55.6) followed by picture of lung disease (32.3%) was found to be most effective. | Continued use of ineffective warning labels on tobacco products represents a missed opportunity as the Government has failed to effectively utilise this evidence-based strategy to enhance knowledge about the effects of tobacco among the people, in addition to other educational interventions, e.g., anti-tobacco advertising employed by the Ministry of Health and Family Welfare in India. |
| Hnin et al 2020 | Myanmar cigarette smokers | May to August 2018 | No data | No data | About 75% intended to reduce the number of cigarettes, and 18% were willing to quit within 6 months. Those who desired to reduce the number of cigarettes were more likely to quit within 6 months (aOR = 7.6, 95% CI 1.6-35.9 and aOR = 19.6, 95% CI 13.0-294.7 for those who had a little and strong desire, respectively. | NR |
| [Creating a smoke-free city – Balanga City, the Philippines](https://www.who.int/news-room/feature-stories/detail/creating-a-smoke-free-city-balanga-city-the-philippines) | NR | NR | NR | NR | A survey showed a decline in adult smoking from 23.8% in 2010 to 14.78% in 2014 and a decline in the prevalence of youth smoking from 32% in 2014 to just 1.63% the following year. There was also a 74% growth in business and a 75.7% increase in tourist arrivals from 2010 to 2017. Since 2012, 22 local government units and three universities have learned from Balanga City’s example and similar ordinances have been adopted by other municipalities. | Unsurprisingly, the city’s actions also received heavy opposition from the tobacco industry. In 2017 the Philippine Tobacco Institute (PTI) challenged both Balanga City’s 2010 ordinance and its 2016 amendment. Unfortunately, a decision from a Regional Trial Court was issued in favour of the industry. A further legal challenge was then filed by PTI in 2018 against the city’s 2016 Tobacco Free Generation Ordinance banning the sale of tobacco products to citizens born after 2000. |
| [Johnson & Johnson donates nicotine patches to help thousands of Jordanians quit tobacco during COVID-19](https://www.who.int/news-room/feature-stories/detail/Johnson-Johnson-donates-nicotine-patches-to-help-thousands-of-jordanians-quit-tobacco-during-COVID-19) | NR | NR | NR | NR | NR | NR |
| [WHO urges more countries to require large, graphic health warnings on tobacco packaging](https://www.who.int/news/item/09-08-2011-who-urges-more-countries-to-require-large-graphic-health-warnings-on-tobacco-packaging) | NR | NR | NR | NR | NR | NR |
| [World governments show unity against tobacco industry and agree to new tobacco-control measures](https://www.who.int/news/item/10-12-2010-world-governments-show-unity-against-tobacco-industry-and-agree-to-new-tobacco-control-measures) | NR | NR | NR | NR | NR | NR |
| [WHO report finds dramatic increase in life-saving tobacco control policies in last decade](https://www.who.int/news/item/19-07-2017-who-report-finds-dramatic-increase-in-life-saving-tobacco-control-policies-in-last-decade) | NR | NR | NR | NR | 43% of the world’s population (3.2 billion people) are covered by two or more MPOWER measures at the highest level, nearly seven times as many people as in 2007. Eight countries, including five low- and middle-income countries, have implemented four or more MPOWER measures at the highest level (Brazil, Islamic Republic of Iran, Ireland, Madagascar, Malta, Panama, Turkey and the United Kingdom of Great Britain and Northern Ireland). | NR |
| [CATCH approach for smokeless tobacco cessation in the South-East Asia region](https://www.who.int/publications/i/item/catch-approach-for-smokeless-tobacco-cessation-in-the-south-east-asia-region) | NR | NR | NR | NR | NR | NR |
| [Hiding Tobacco Products at Convenience Stores Reduces Teens' Risk of Future Tobacco Use](https://www.rand.org/news/press/2015/11/23.html) | NR | NR | NR | NR | Researchers found hiding the tobacco power wall significantly reduced teenagers' susceptibility to future cigarette smoking compared to leaving the tobacco advertising visible. Moving the power wall to a less-obvious location did not have any effect.  Researchers say the results provide information that the federal Food and Drug Administration's Center for Tobacco Products could potentially use to make future regulatory decisions for point-of-sale tobacco advertising. | NR |
| [Graphic Warning Labels on Tobacco Packages Can Deter Some Smokers from Buying Cigarettes](https://www.rand.org/news/press/2019/04/09.html) | NR | NR | NR | NR | NR | NR |
| [Reducing Teen Smoking and Drinking through Increased Schooling in the Dominican Republic](https://www.povertyactionlab.org/evaluation/reducing-teen-smoking-and-drinking-through-increased-schooling-dominican-republic) | NR | NR | NR | NR | NR | NR |

**Supplementary table 6: Details on Funding**

| **Study ID** | **Funding received (Yes/No)** | **Name of funding agency** | **Role of funding agency (Design, implementation, analysis)** | **Conflict of interest reported (Yes/No)** | **Country where the intervention was delivered** |
| --- | --- | --- | --- | --- | --- |
| Ward et al 2006 | Yes | Tobacco Research and capacity Building Program” (USPHS, R01TW05962). A start-up grant (SUG) for tobacco related research from the Initiative for Cardiovascular Health Research in the Developing Countries (IC-Health), and a grant from the “Health, Environment, and Economic Development” program (R21 TW006545). | The Syrian Center for Tobacco Studies was established by a grant of the Fogarty International Center's “Tobacco Research and capacity Building Program” (USPHS, R01TW05962). | Yes, None | Syria |
| Ayub et al 2015 | Yes | KHCC is a grantee of the Mayo Clinic (USA) for conducting Global Bridges work in the Eastern Mediterranean Region. Development of the guidelines was funded in part by this grant. | NR | Yes, None | Jordan |
| Uang et al 2017 | Yes | This project was supported in part by National Cancer Institute grant CA-087472 and UCSF funds from the FAMRI William Cahan Endowment Fund and Dr. Glantz’ American Legacy Foundation Distinguished Professorship. | The funding agencies played no role in the selection of the research question, conduct of the research, or preparation of the manuscript. | Yes, None | Colombia |
| Bader et al 2017 | Yes | World Health Organization – Jordan Country Office. | NR | Yes, None | NR |
| Odukoya et al 2016 | Yes | Pfizer Independent Grants for learning and change. Grant ID: 13509341. | NR | Yes, None | Nigeria |
| Asare et al 2019 | No | NA | NA | Yes, None | NA |
| Maldonado 2022 | Yes | Global Tobacco Economics Consortium GTEC project. Funded by IDRC (Grant 108819) | NA | Yes, None | Colombia |
| Andreeva et al 2011 | Yes | Campaign for Tobacco Free Kids within the Bloomberg Global Initiative to reduce tobacco use | The data collection was performed by the Kyiv International Institute of Sociology and supported with funds from the Campaign for Tobacco Free Kids within the Bloomberg Global Initiative to reduce tobacco use. | Yes, None | Ukraine |
| Egbe 2018 | Yes | This work was funded by National Cancer Institute Grant CA-087472. | The funding agency played no role in the conduct of the research or preparation of the manuscript | Yes, None | Nigeria |
| Adebiyi 2016 | No | NA | NA | Yes, None | Nigeria |
| Perl et al 2014 | Yes | Bloomberg Philanthropies and the Bill and Melinda Gates Foundation | not given | Yes, None | Senegal, Nigeria and Kenya |
| Hussain et al 2018 | No | NA | NA | Yes, None | Pakistan |
| Hallit et al 2019 | No | NA | NA | Yes, None | Lebanon |
| Mapa-Tassou 2018 | Yes | The Analysis of Non-Communicable Disease Policies in Africa study was funded by the International Development Research Centre (grant # 107209– 001) through the Africa Population and Health Research Center in Nairobi, Kenya. Publication charges were paid by the International Development Research Centre | NR | Yes, None | Cameroon |
| Girvalaki et al 2020 | Yes | Global Bridges: Healthcare Alliance for Tobacco Dependence Treatment; and a Pfizer Independent Grants for Learning and Change (GB- 25944945). | NA | Yes, None | Albania, Armenia, Belgium, Italy, France, Georgia, Greece, Kosovo, Romania, North Macedonia, Russia, Serbia, Slovenia, Spain, Ukraine |
| Chopra et al 2014 | No | NA | NA | Yes, None | Barwala, Panchkula, Haryana, India |
| Hnin et al 2020 | Yes | Ministry of Health and Sports, Department of Medical Research, Yangon, Myanmar: Implementation Research Funding Grant ID: 24/2018. | NR | Yes, None | Mayanmar |
| [Creating a smoke-free city – Balanga City, the Philippines](https://www.who.int/news-room/feature-stories/detail/creating-a-smoke-free-city-balanga-city-the-philippines) | NR | NR | NR | NR | Philippines |
| [Johnson & Johnson donates nicotine patches to help thousands of Jordanians quit tobacco during COVID-19](https://www.who.int/news-room/feature-stories/detail/Johnson-Johnson-donates-nicotine-patches-to-help-thousands-of-jordanians-quit-tobacco-during-COVID-19) | Yes | Johnson & Johnson | Implementation | NR | Jordan |
| [WHO urges more countries to require large, graphic health warnings on tobacco packaging](https://www.who.int/news/item/09-08-2011-who-urges-more-countries-to-require-large-graphic-health-warnings-on-tobacco-packaging) | NR | NR | NR | NR | Multiple |
| [World governments show unity against tobacco industry and agree to new tobacco-control measures](https://www.who.int/news/item/10-12-2010-world-governments-show-unity-against-tobacco-industry-and-agree-to-new-tobacco-control-measures) | NR | NA | NA | NR | Multiple |
| [WHO report finds dramatic increase in life-saving tobacco control policies in last decade](https://www.who.int/news/item/19-07-2017-who-report-finds-dramatic-increase-in-life-saving-tobacco-control-policies-in-last-decade) | NR | NA | NA | NR | Multiple |
| [CATCH approach for smokeless tobacco cessation in the South-East Asia region](https://www.who.int/publications/i/item/catch-approach-for-smokeless-tobacco-cessation-in-the-south-east-asia-region) | NR | NA | NA | NR | SEA region |
| [Hiding Tobacco Products at Convenience Stores Reduces Teens' Risk of Future Tobacco Use](https://www.rand.org/news/press/2015/11/23.html) | Yes | National Cancer Institute and FDA Center for Tobacco Products | NA | NR | US |
| [Graphic Warning Labels on Tobacco Packages Can Deter Some Smokers from Buying Cigarettes](https://www.rand.org/news/press/2019/04/09.html) | Yes | RAND Social and Economic Well-Being | NA | NR | US |
| [Reducing Teen Smoking and Drinking through Increased Schooling in the Dominican Republic](https://www.povertyactionlab.org/evaluation/reducing-teen-smoking-and-drinking-through-increased-schooling-dominican-republic) | No | NA | NA | NR | Dominican Republic |
